# Effects of Melatonin-Loaded Poly(N-vinylcaprolactam) Transdermal Gel on Sleep Quality

**DOI:** 10.3390/gels11060435

**Published:** 2025-06-05

**Authors:** Wei Zhao, Fengyu Wang, Liying Huang, Bo Song, Junzi Wu, Yongbo Zhang, Wuyi Du, Yan Li, Sen Tong

**Affiliations:** 1 Faculty of Life Science and Technology, Kunming University of Science and Technology, Kunming 650500, China; zhaowei@ynucm.edu.cn; 2 Yunnan Key Laboratory of Integrated Traditional Chinese and Western Medicine for Chronic Disease in Prevention and Treatment, Yunnan University of Chinese Medicine, Kunming 650500, China; wangfengyu@ynucm.edu.cn (F.W.); huangliying1609@163.com (L.H.); ynkmsongbo6@126.com (B.S.); xnfz@ynucm.edu.cn (J.W.); nameiszhang6896@163.com (Y.Z.); 3 Department of Geriatrics, The First People’s Hospital of Yunnan Province, Kunming 650032, China; 15587201738@163.com; 4 Medical School, Kunming University of Science and Technology, Kunming 650500, China

**Keywords:** melatonin, poly(N-vinylcaprolactam), transdermal gel, sleep quality, transdermal permeation enhancers

## Abstract

The rapid pace of modern life has contributed to a significant decline in sleep quality, which has become an urgent global public health issue. Melatonin, an endogenous hormone that regulates circadian rhythms, is vital in maintaining normal sleep cycles. While oral melatonin supplementation is widely used, transdermal delivery systems present advantages that include the avoidance of first-pass metabolism effects and enhanced bioavailability. In this study, a novel melatonin transdermal delivery system was successfully developed using a thermosensitive poly(N-vinylcaprolactam) [p(NVCL)]-based carrier. The p(NVCL) polymer was synthesized through free radical polymerization and characterized for its structural properties and phase transition temperature, in alignment with skin surface conditions. Orthogonal optimization experiments identified 3% azone, 3% menthol, and 4% borneol as the optimal enhancer combination for enhanced transdermal absorption. The formulation demonstrated exceptional melatonin loading characteristics with high encapsulation efficiency and stable physicochemical properties, including an appropriate pH and optimal moisture content. Comprehensive in vivo evaluation using normal mouse models revealed significant sleep quality improvements, specifically a shortened sleep latency and extended non-rapid eye movement sleep duration, with elevated serum melatonin and serotonin levels. Safety assessments including histopathological examination, biochemical analysis, and 28-day continuous administration studies confirmed excellent biocompatibility with no adverse reactions or systemic toxicity. Near-infrared fluorescence imaging provided direct evidence of enhanced transdermal absorption and superior biodistribution compared to oral administration. These findings indicate that the p(NVCL)-based melatonin transdermal gel system offers a safe, effective and convenient non-prescription option for sleep regulation, with promising potential for clinical translation as a consumer sleep aid.

## 1. Introduction

Sleep is a fundamental activity for maintaining physiological functions and is irreplaceable in the recovery of higher neural systems as well as overall physical and mental health [1]. In the context of accelerating globalization and rising social pressures, sleep quality issues have become a significant public health challenge [2,3]. Epidemiological surveys have shown that sleep disturbances are prevalent in major developed countries globally, with approximately 24.8% of the adult population in China experiencing poor sleep quality [4]. The impact of poor sleep quality is multifaceted: cognitively, it can lead to memory decline, attention deficits and impaired decision making [5]; physiologically, long-term sleep deprivation is associated with immune dysfunction and may increase the risk of cardiovascular and cerebrovascular diseases [6]; and socially, individuals with poor sleep quality face a 2.5–3.0-times higher risk of traffic accidents than those with good sleep [7]. Given the severity and widespread impact of sleep quality issues, exploring effective methods for improvement is crucial. Current approaches for improving sleep quality primarily include non-pharmacological interventions and pharmacological support. Although non-pharmacological interventions exhibit better long-term efficacy, their practical application remains limited due to insufficient professional guidance and the delayed onset of effects [8,9]. Conversely, synthetic hypnotic medications provide rapid relief but are associated with tolerance, dependence and various adverse reactions [10]. Therefore, developing safe, effective and well-tolerated natural agents for regulating sleep quality has significant clinical value and social relevance.

Melatonin, a key endogenous hormone that regulates circadian rhythms and is secreted by the pineal gland, is crucial in maintaining sleep–wake cycles and improving sleep quality [11]. A substantial body of research shows that the appropriate supplementation of exogenous melatonin not only effectively shortens sleep latency and improves overall sleep quality but also does not induce drug dependence, with safety profiles superior to traditional hypnotic medications. However, most currently available melatonin preparations are administered orally, a route associated with significant first-pass metabolism effects [12]. Furthermore, melatonin has a relatively short biological half-life in vivo, which makes it challenging to maintain stable, effective serum concentrations, thereby limiting its prolonged effects on sleep quality [13]. To address these limitations, transdermal delivery systems present several advantages, including the avoidance of first-pass metabolism effects, convenient administration and controllable release mechanisms, thereby providing new avenues for research and development aimed at improving the bioavailability of melatonin and enhancing its pharmacological duration [14]. Compared with traditional patches, gel formulations enable us to flexibly adjust the administration area and dosage based on individual needs. In addition, they provide an improved user experience and enhance patient compliance [15]. Previous research has explored alternative gel formulations for melatonin delivery. Laohasiriwong et al. demonstrated that melatonin niosome gel applied to upper labial mucosa could effectively induce daytime sleep in healthy volunteers with a satisfactory sleep duration and onset latency [16].

The selection of appropriate carrier materials is crucial for developing efficient transdermal delivery systems. Poly(N-vinylcaprolactam) [p(NVCL)], a novel intelligent responsive polymer, offers unique advantages in overcoming the challenges outlined above [17]. First, p(NVCL) exhibits excellent biocompatibility, effectively minimizing skin irritation, and is suitable for long-term transdermal application [18]. Second, compared with traditional thermosensitive materials, such as poly(N-isopropylacrylamide), the phase transition temperature of p(NVCL) more closely aligns with the human skin surface temperature, allowing for a precise response under physiological conditions [19]. From a structure–function relationship perspective, the favorable molecular characteristics of p(NVCL) facilitate the formation of physical adsorption complexes with melatonin molecules, thereby improving the melatonin loading capacity of the formulation [20]. More importantly, the thermosensitive response properties of p(NVCL) enable a controlled phase transition from sol to gel under skin surface temperature conditions. This transition forms appropriate drug micro-reservoirs, creating an ideal microenvironment for the sustained release and efficient transdermal absorption of melatonin molecules. Consequently, these properties overcome the limitations of oral administration and extend the duration of drug action [21]. However, to our knowledge, no previous studies have specifically investigated p(NVCL) as a carrier material for melatonin transdermal delivery systems.

Building upon the above theoretical foundation, we selected p(NVCL) as the primary matrix material for developing a novel melatonin transdermal delivery system. p(NVCL) polymer, suitable for transdermal application, was synthesized using free radical polymerization, and its phase transition temperature, molecular weight distribution and structural properties were systematically characterized. A gel-based formulation that exhibited good rheological properties, thixotropy and a well-defined microstructure was then developed. To enhance drug transdermal absorption efficiency, we initially screened the permeation enhancement effects of various single transdermal enhancers (azone, menthol, and borneol). Since multiple permeation enhancers required simultaneous optimization at various concentration levels, we employed orthogonal experimental design, ultimately establishing a highly efficient composite enhancer system that significantly improved the transdermal absorption performance of melatonin. In normal mouse models, the safety and sleep quality improvement effects of this gel formulation were systematically evaluated through a comprehensive evaluation system, which included behavioral experiments, electroencephalogram monitoring and biochemical marker analysis. Overall, this study not only broadens the application of p(NVCL) in functional drug delivery systems but also provides an innovative technological platform and a solid theoretical foundation for developing safe, effective and convenient melatonin-based healthcare products.

## 2. Results and Discussion

### 2.1. Preparation and Characterisation of Melatonin-Loaded p(NVCL) Transdermal Gel

#### 2.1.1. Polymer Synthesis Results and Structural Confirmation

p(NVCL) was successfully synthesized using 2,2′-azobis[2-(2-imidazolin-2-yl)propane] dihydrochloride (AIBI) as an initiator through free radical polymerization. The resulting white powder product was obtained with a yield of 83.3%, comparable to similar synthesis methods using the AIBN initiator, as reported in the literature [22]. This synthesis method is straightforward, requires no complex post-processing procedures and is suitable for scale-up production. The selection of p(NVCL) as the melatonin carrier material was based on several key scientific considerations, including its superior biosafety profile compared to traditional thermosensitive polymers, optimal phase transition temperature alignment with skin surface conditions, enhanced chemical stability, and favorable molecular characteristics for drug loading [23].

**Figure 1:**
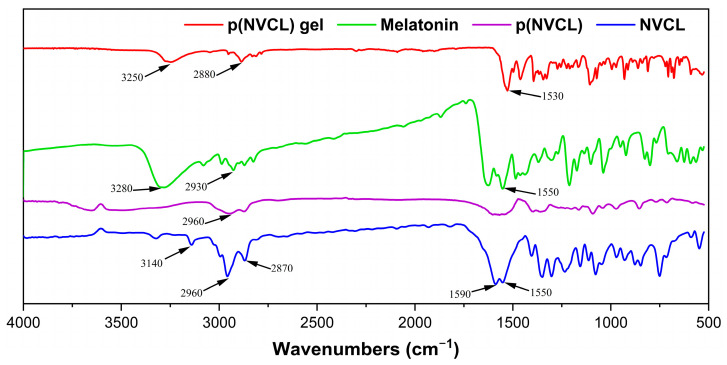
FTIR spectra comparison of NVCL monomer, p(NVCL) polymer, melatonin, and melatonin-loaded p(NVCL) gel.

The chemical structure and successful drug incorporation were confirmed through a comprehensive FTIR spectroscopy analysis of all components (Figure 1). The NVCL monomer exhibited characteristic peaks including C=C stretching (1590 cm^−1^), C=O stretching (1550 cm^−1^), CH_2_ stretching (2870 and 2960 cm^−1^) and C=CH stretching (3140 cm^−1^). The p(NVCL) polymer spectrum demonstrated a complete absence of the C=C stretching peak at 1590 cm^−1^, confirming successful polymerization. The polymer retained the CH_2_ stretching vibration at 2960 cm^−1^, consistent with the established p(NVCL) characteristics reported in previous studies [24].

Pure melatonin exhibited distinctive FTIR characteristics, with key absorption bands at 3280 cm^−1^ corresponding to N-H stretching vibrations from the indole moiety, CH_2_ stretching at 2930 cm^−1^, and C=O stretching at 1550 cm^−1^. These peaks represent the fundamental molecular structure of melatonin and serve as reference markers for evaluating drug incorporation. The melatonin-loaded gel formulation revealed distinctive spectroscopic features that provided direct evidence of drug–polymer interactions and successful incorporation. Most notably, the gel spectrum showed a characteristic peak at 3250 cm^−1^, representing a slight shift from the pure melatonin N-H stretching vibration (3280 cm^−1^), indicating molecular interactions between melatonin and the p(NVCL) carrier system. The gel formulation also exhibited CH_2_ stretching at 2880 cm^−1^ and C=O stretching at 1530 cm^−1^. These peak shifts and broadening effects provided spectroscopic evidence for the formation of intermolecular interactions between melatonin molecules and the polymer backbone through hydrogen bonding, confirming successful drug loading within the p(NVCL) carrier system.

#### 2.1.2. Thermal Properties and Molecular Weight Distribution

The thermosensitive behaviour of the synthesized p(NVCL) was assessed through differential scanning calorimetry (DSC) analysis. The lower critical solution temperature (LCST) was found to be 33.5 °C, which is particularly favorable for transdermal applications due to its close alignment with the skin surface temperature. This optimal LCST ensures that the polymer undergoes a phase transition precisely at the application site, potentially facilitating efficient temperature-responsive control over drug release [25]. The molecular weight distribution of p(NVCL) was analyzed through gel permeation chromatography (GPC), as illustrated in Figure S1. The weight-average molecular weight (M_w_) was 4.6 × 10^4^ g/mol, whereas the number-average molecular weight (M_n_) was 2.5 × 10^4^ g/mol, yielding a polydispersity index (PDI) of 1.86. This relatively narrow molecular weight distribution is favorable for the formation of a uniform drug-loaded network structure with consistent mechanical and release properties.

#### 2.1.3. Melatonin Loading and Encapsulation Characteristics

Following the successful synthesis and Characterisation of p(NVCL) as an ideal carrier material, our research focused on optimizing the formulation process to maximize the melatonin loading efficiency. Several technical challenges were encountered during the Optimisation process, primarily revolving around balancing the unique swelling characteristics of p(NVCL), the dynamics of network formation and the need for the uniform dispersion of melatonin throughout the matrix. Initial formulation attempts involving the direct co-dispersion of p(NVCL) and melatonin in an aqueous medium resulted in significant drug aggregation and inconsistent loading. A subsequent approach employing an organic solvent-assisted method improved the uniformity of drug dispersion; however, it resulted in residual solvent levels that exceeded pharmacopeial thresholds for transdermal applications. These preliminary outcomes necessitated a comprehensive reformulation of the preparation methodology. Following a systematic evaluation of multiple formulation variables, an optimized two-step process was established. In this refined approach, the primary p(NVCL) network structure was initially formed under strictly controlled low-temperature conditions (4 °C), after which hydroxypropyl methylcellulose (HPMC) and Carbopol were gradually incorporated under carefully regulated temperature gradients. This methodological advancement significantly enhanced the structural uniformity of the polymer network and consistent distribution of melatonin throughout the matrix.

The optimized formulation exhibited exceptional melatonin loading characteristics, with a melatonin content of 2.36 ± 0.12% and an encapsulation efficiency of 78.7 ± 4.0%, determined using the formula encapsulation efficiency = (measured melatonin mass/theoretical melatonin content) × 100, with a theoretical melatonin content of 3% (*w*/*w*). Each gram of the gel formulation reliably delivered 23.6 mg of melatonin, representing a therapeutically relevant dose appropriate for transdermal administration. The high encapsulation efficiency was primarily attributed to favorable molecular interactions between melatonin and the p(NVCL) polymer matrix, particularly the formation of multiple non-covalent bonds. These included hydrogen bonding between the amide groups of p(NVCL) and the indole nitrogen of melatonin, as well as hydrophobic interactions between the polymer backbone and the aromatic regions of the drug molecule [26,27].

#### 2.1.4. Powder X-Ray Diffraction Analysis and Crystalline State Characterization

**Figure 2:**
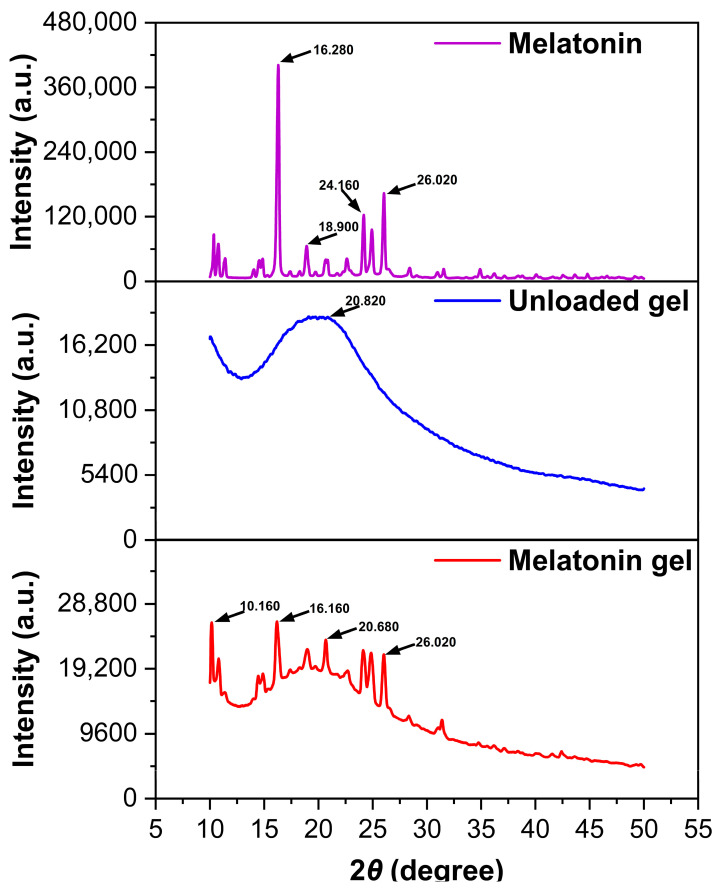
Powder X-ray diffraction (PXRD) patterns comparing pure melatonin, melatonin-loaded gel, and unloaded gel.

PXRD analysis provided valuable insights into the crystalline characteristics and molecular interactions within the formulation system (Figure 2). Pure melatonin exhibited sharp, well-defined diffraction peaks at 16.280°, 26.020°, 24.160°, and 18.900° with high intensities, confirming the highly crystalline nature of the raw material. The p(NVCL) polymer displayed a characteristic broad peak at 20.820° with wide half-width, indicating the predominantly amorphous nature of the polymer carrier. The melatonin-loaded gel formulation showed multiple diffraction peaks at 16.160°, 10.160°, 20.680°, and 26.020°, with notably reduced intensities compared to pure melatonin. The presence of melatonin characteristic peaks in the final formulation confirmed successful drug incorporation, while the significant intensity reduction indicated partial conversion from a crystalline to amorphous state during the encapsulation process. The PXRD results demonstrated that the formulation process led to a reduction in melatonin crystallinity, which supports the enhanced dissolution and encapsulation efficiency observed in previous characterizations. The coexistence of both crystalline and amorphous characteristics in the final formulation suggests an optimal drug distribution within the polymer network, providing the controlled release properties essential for transdermal delivery applications.

### 2.2. Physicochemical Properties of Melatonin-Loaded p(NVCL) Transdermal Gel

**Figure 3:**
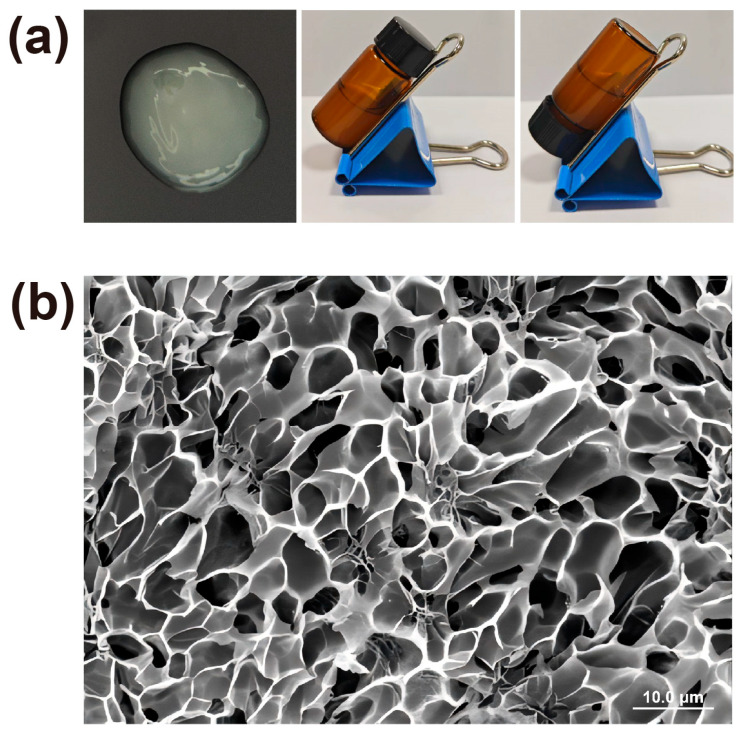
Characterization of melatonin-loaded p(NVCL) transdermal gel. (**a**) Physical appearance showing translucent, homogeneous gel formulation. (**b**) Cryo-SEM micrograph at 1500× magnification revealing microporous network structure.

The resulting melatonin-loaded p(NVCL) transdermal gel appeared as a translucent and homogeneous formulation, exhibiting excellent physical stability with no evidence of phase separation. Physicochemical analysis revealed a pH of 6.8. The gel exhibited a moisture content of 75.3 ± 1.4%, which is conducive to maintaining skin hydration and promoting enhanced drug permeation through the stratum corneum. Microstructural analysis using cryogenic scanning electron microscopy (Cryo-SEM) provided valuable insights into the internal architecture of the gel. As illustrated in Figure 3 (×1.50 k magnification), the formulation exhibited a semi-continuous membrane-like morphology, characterized by a network of irregularly distributed micropores and cavities throughout the surface. These microporous structures, measuring approximately 10–20 μm in diameter, were distributed throughout the gel matrix. Quantitative analysis using ImageJ software (version 1.54p) revealed that the gel matrix possessed a porosity of 45.95%, indicating a highly porous network structure that facilitates drug diffusion and release. Notably, certain pores contained visible particulate matter, which were suggested to be melatonin crystals incorporated within the polymer network through microscopic analysis [28]. This unique microstructure plays a crucial role in determining both the mechanical properties and drug release kinetics of the formulation.

### 2.3. Optimisation of Transdermal Permeation Enhancers Transdermal Permeation Enhancement Performance

#### 2.3.1. Screening of Single Permeation Enhancers Single Enhancer Efficacy Results

**Figure 4:**
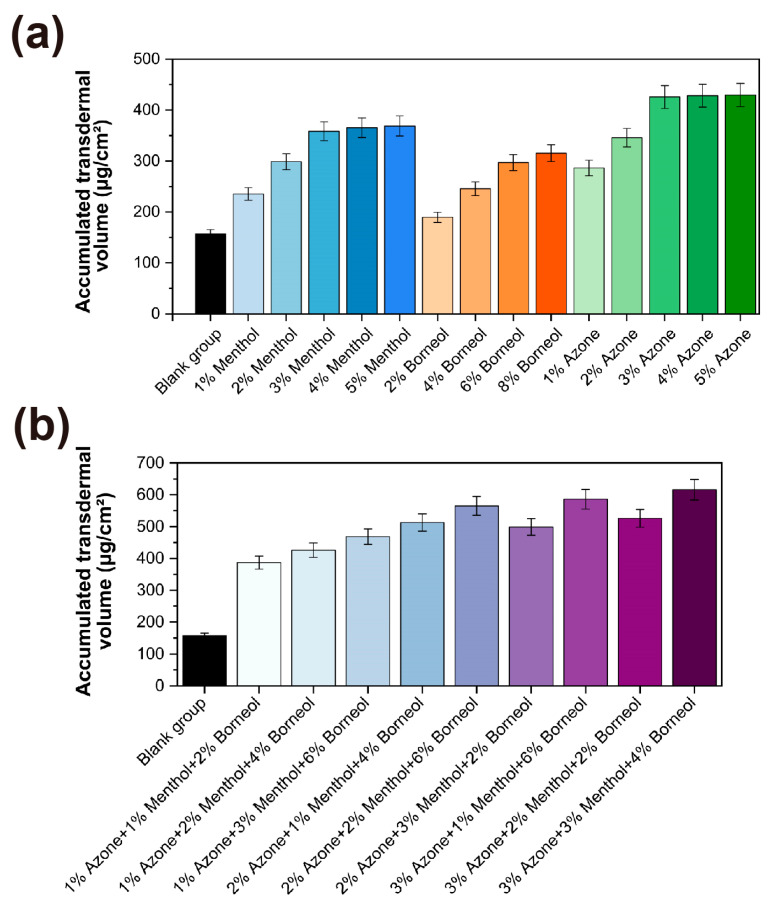
Effects and optimization of transdermal penetration enhancers on the cumulative permeation of melatonin. (**a**) Effects of single transdermal penetration enhancers (azone, menthol and borneol) at varying concentrations on the cumulative permeation of melatonin; (**b**) Evaluation of different combinations of three transdermal penetration enhancers using L9(3^3^) orthogonal experimental design on the 24 h cumulative permeation of melatonin.

Melatonin was chosen as the active ingredient in this formulation due to its function as an endogenous sleep-regulating hormone that facilitates sleep initiation while preserving the physiological sleep structure without causing dependence during prolonged use [29]. Based on the various mechanisms of transdermal enhancement reported in the literature, this study evaluated azone, borneol and menthol as potential permeation enhancers using a modified Franz diffusion cell system with excised rat skin [30]. In the control formulation without enhancers, the cumulative permeation over 24 h was 156.8 ± 8.5 μg/cm^2^ with a corresponding transdermal flux of 6.53 ± 0.35 μg/cm^2^/h. All three enhancers exhibited concentration-dependent effects (Figure 4a). Menthol showed enhanced permeation within the 1–3% (*w*/*w*) range, reaching a peak of 358.2 ± 18.4 μg/cm^2^ at 3% concentration, with no significant additional benefit observed at higher concentrations. Borneol exhibited moderate enhancement, reaching 315.4 ± 16.5 μg/cm^2^, even at an 8% concentration. Azone exhibited the most significant effect, reaching 425.6 ± 22.4 μg/cm^2^ at 3% concentration, with a stable performance maintained in the concentration range of 3–5%. Among single enhancers, azone demonstrated the highest transdermal flux of 17.73 ± 0.93 μg/cm^2^/h at the optimal concentration (Table S2). These findings are consistent with previous research highlighting the superior ability of azone to disrupt stratum corneum lipid structures compared with terpene-based enhancers [31].

#### 2.3.2. Composite Enhancer System Performance Evaluation

To fully harness the pharmacological advantage of melatonin, a multi-target stratum for enhancing corneum penetration was designed, rather than relying on simple empirical combinations. Based on the results of the single enhancer screening, an L9(3^3^) orthogonal experimental design was employed to identify the optimal enhancer combination with fewer experimental variables. The transdermal parameters for each experimental combination are summarized in Figure 4b. Statistical analysis revealed that the composite enhancer system, consisting of 3% azone, 3% menthol and 4% borneol, exhibited superior enhancement effects compared with any single enhancer formulation. This optimized combination achieved a 24 h cumulative permeation of 615.8 ± 32.4 μg/cm^2^, representing a 3.9-fold increase relative to the control group. The corresponding transdermal flux reached 25.66 ± 1.35 μg/cm^2^/h (Table S2). The exceptional performance of this composite system can be attributed to the complementary molecular mechanisms through which each enhancer functions. Azone selectively integrates into the intercellular lipid bilayers of the stratum corneum, disrupting tight packing and enhancing fluidity. Menthol primarily interacts with the polar head group region of stratum corneum lipids, forming hydrogen bond networks that temporarily reorganize lipid arrangements while significantly increasing the partition coefficient of melatonin between lipids. Borneol exerts a dual action on both the lipid and protein domains of the stratum corneum, forming temporary permeable channels [32,33,34]. Through this multi-target synergistic action, a significant enhancement in the melatonin transdermal absorption efficiency was achieved while minimizing the risk of skin irritation, which could be caused by high concentrations of single enhancers. This optimization is particularly beneficial for populations such as older adults, who experience a decline in physiological melatonin secretion, and shift workers, who require adjustments to their sleep–wake cycles [35,36]. The optimized composite enhancer system provides a solid foundation for subsequent studies on the effects of the melatonin-loaded p(NVCL) transdermal gel on sleep quality in mice.

### 2.4. Physical–Mechanical Properties of Melatonin-Loaded p(NVCL) Transdermal Gel

#### 2.4.1. Rheological Property Analysis

**Figure 5:**
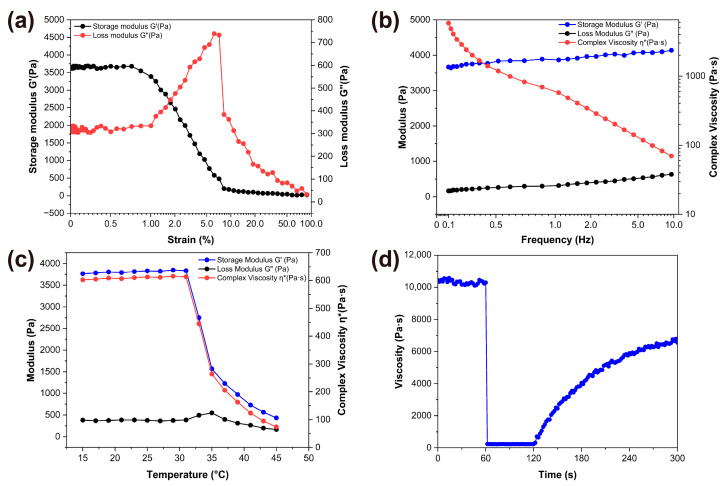
Rheological and thixotropic properties of melatonin-loaded p(NVCL) transdermal gel. (**a**) Strain sweep analysis showing storage modulus (G′) and loss modulus (G″) versus strain percentage; (**b**) Frequency sweep analysis displaying storage modulus (G′) and loss modulus (G″) versus frequency (0.1–10 Hz); (**c**) Temperature sweep analysis showing storage modulus (G′) and loss modulus (G″) versus temperature (15–45 °C); (**d**) Thixotropic behavior evaluation showing viscosity changes during three-step shear testing over time.

Strain sweep experiments revealed that the melatonin-loaded p(NVCL) transdermal gel exhibited good structural stability. Beyond a critical strain point, the storage modulus (G′) value decreased rapidly, indicating the breakdown of the gel network structure (Figure 5a). In the frequency sweep test, across the entire test frequency range (0.1–10 Hz), the G′ consistently exceeded the loss modulus (G″), indicating typical gel behaviour (Figure 5b). At 0.1 Hz, the G′ value was 3650 Pa, increasing slightly to 4120 Pa at 10 Hz, indicating a robust network structure. The temperature sweep results showed that the gel maintained structural stability between 15 °C and 32 °C, with a significant transition occurring between 32 °C and 34 °C, where the G′ value decreased from 3810 Pa to 1770 Pa (Figure 5c). This transition point, which is close to skin temperature, is particularly beneficial for transdermal delivery systems [17].

#### 2.4.2. Thixotropic Property Evaluation

The three-step shear test results indicated that the gel exhibited high viscosity (10,550 Pa·s) during the initial low shear phase (0.1 s^−1^), which rapidly decreased to 235 Pa·s under high shear force (100 s^−1^) before gradually recovering to 7205 Pa·s during the recovery phase (Figure 5d). This behaviour indicated that the gel possessed good structural recoverability, making it suitable for skin application. Texture analysis revealed that the gel extended to a distance of 15.5 mm, indicating moderate extensibility without any noticeable breaking or separation, which is beneficial for uniform application on the skin. A comprehensive analysis revealed that the prepared melatonin-loaded p(NVCL) transdermal gel exhibited optimal viscosity and flowability. It maintained structural stability at room temperature while sufficiently softening at skin temperature to facilitate drug release. Furthermore, the gel exhibited good thixotropic properties that met the requirements of transdermal delivery systems [37].

#### 2.4.3. Stability Studies Formulation Stability Assessment Results

The stability of the optimized formulation was evaluated over a 6-month period under three distinct storage conditions (Table S1). The results showed that the gel retained its stability under room temperature (25 ± 2 °C) and low-temperature (4 ± 2 °C) conditions. Throughout the study period, the formulation maintained a transparent, homogeneous appearance with no observable phase separation or turbidity, indicating the structural integrity of the p(NVCL) network. Minimal variations were observed in physicochemical indicators, such as pH, viscosity and extensibility. Furthermore, the drug content met the quality standard requirements (retention rate > 90%) across all storage conditions. Under accelerated testing conditions, a marginal decline in specific indicators was observed; however, the drug retention rate at 6 months remained high (93.2 ± 1.6%), affirming the good stability of the formulation.

### 2.5. In Vivo Evaluation of Sleep Quality Improvement and Safety of Melatonin-Loaded p(NVCL) Transdermal Gel

After identifying the optimal carrier material and optimizing the transdermal enhancer formulation, the physiological mechanisms through which melatonin-loaded p(NVCL) transdermal gel improved sleep quality were explored. The methodological approach was designed to progress systematically, assessing observable behavioral changes, conducting a detailed sleep architecture analysis and finally elucidating the underlying biochemical mechanisms.

#### 2.5.1. Behavioral Assessment of Sleep Promotion Sleep Promotion Behavioral Outcomes

**Figure 6:**
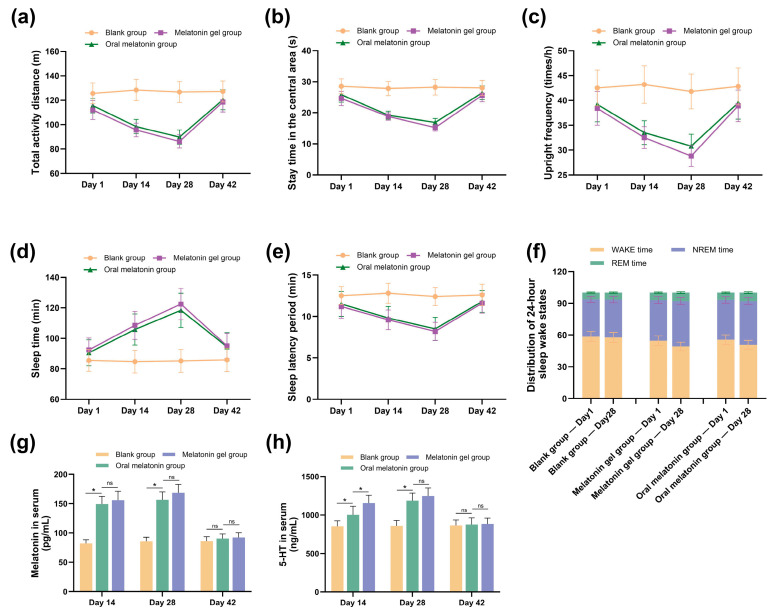
Comparison of the effects of melatonin gel, oral melatonin administration and a blank group on sleep parameters in mice. Data are expressed as mean ± standard deviation. (**a**–**c**) Behavioral parameters measured using 12 mice per group: (**a**) total activity distance, (**b**) time spent in the central area, (**c**) upright frequency; (**d**,**e**) pentobarbital-induced sleep test parameters using the same 12 mice per group: (**d**) total sleep time, (**e**) sleep latency period; (**f**) sleep–wake state distribution showing wakefulness (WAKE) time, NREM time, and rapid eye movement (REM) time derived from 24 h EEG recordings on day 28 using the same 12 mice per group; (**g**,**h**) serum biochemical indicators measured using six mice per group at each timepoint: (**g**) serum melatonin levels and (**h**) serum 5-HT levels, measured on days 14, 28 and 42. Statistical significance notation across all figures: * *p* < 0.05; ns = not significant.

Behavioral assessment was employed as the initial platform for evaluation. It revealed that mice treated with the melatonin gel exhibited a significant reduction in motor activity, reflecting the inhibitory effects of melatonin on arousal systems [38]. As shown in Figure 6a–c, compared with the blank group, mice in the melatonin gel group exhibited a significant decrease in the total activity distance, a reduced amount of time spent in the central area, and fewer instances of rearing during the 6 h observation period following administration (*p* < 0.05). These changes in behavioral parameters were consistent with the central inhibitory effects of melatonin, as reported by Nasini et al., indicating that the melatonin in the gel formulation successfully penetrated the skin barrier and exerted regulatory effects on the central nervous system [39]. In the pentobarbital-induced sleep test (Figure 6d,e), the melatonin gel group exhibited a significant reduction in sleep latency and a prolonged sleep duration (*p* < 0.05), further confirming effective melatonin absorption [40]. Notably, these effects were comparable to those observed in the oral melatonin group. However, the transdermal administration group exhibited more consistent effects following long-term application (28 days), likely due to the more uniform and stable drug release profile associated with the transdermal delivery system. Consistent with the findings of Hu et al., our study showed that the efficacy of the melatonin treatment remained stable throughout the 28-day continuous administration period, with no apparent development of tolerance. This contrasts sharply with the efficacy reduction commonly observed with traditional hypnotic drugs, such as benzodiazepines, during long-term use [41]. Furthermore, behavioral indicators returned to baseline levels 14 days after the cessation of treatment, indicating the absence of drug dependence or residual effects. This supports the safety profile of the formulation, highlighting its suitability for long-term use.

#### 2.5.2. Electroencephalographic Sleep Structure Analysis EEG-Based Sleep Architecture Analysis Results

In addition to behavioral observations, our electroencephalographic analysis provided more definitive evidence for sleep quality improvement. Continuous 24 h EEG recordings on day 28 of treatment (Figure 6f) showed that the melatonin-loaded p(NVCL) transdermal gel significantly improved sleep structure parameters in mice. Compared with the blank group, mice in the melatonin gel group exhibited a notably extended average duration of continuous non-rapid eye movement (NREM) sleep episodes (*p* < 0.05). This improvement in sleep continuity was identified as a key indicator of improved sleep quality by Wang et al. [42]. A comparative analysis between the transdermal gel and oral melatonin groups revealed that the p(NVCL) melatonin-loaded transdermal gel had a more significant effect on NREM sleep proportion, a finding consistent with that of Sevilla et al., who indicated that stable melatonin blood concentrations are more effective in improving deep sleep [43].

#### 2.5.3. Neurochemical Mechanism Evaluation

In this study, we quantitatively analyzed serum levels of melatonin and 5-hydroxytryptamine (5-HT) using ELISA (Figure 6g,h). The results demonstrate that both the oral melatonin group and melatonin gel group showed significantly elevated serum melatonin and 5-HT levels compared to the blank group at day 14 (*p* < 0.05). After 28 days of treatment, the melatonin gel group exhibited significantly higher serum melatonin levels than the blank group (*p* < 0.05). Simultaneously, the serum 5-HT levels in the melatonin gel group were significantly elevated, consistent with the findings of Xia et al., who investigated the ability of melatonin to modulate 5-HT neuronal activity [44]. The increase in 5-HT levels may be an important neurochemical basis for NREM sleep improvement, as 5-HT promotes deep sleep by regulating hypothalamic sleep centers. A key safety finding was that both melatonin and 5-HT levels returned to baseline 14 days after treatment cessation, indicating no long-term disruption of endogenous regulatory mechanisms. This represents a significant advantage over many traditional sleep medications, which often lead to tolerance or dependence, as extensively documented in the clinical literature [45]. These findings are consistent with the findings of Givler et al., who indicated that exogenous melatonin supplementation does not result in the long-term inhibition of endogenous melatonin secretion, supporting its potential for safe long-term use as a sleep regulator [29].

#### 2.5.4. Safety and Tolerability Evaluation

**Figure 7:**
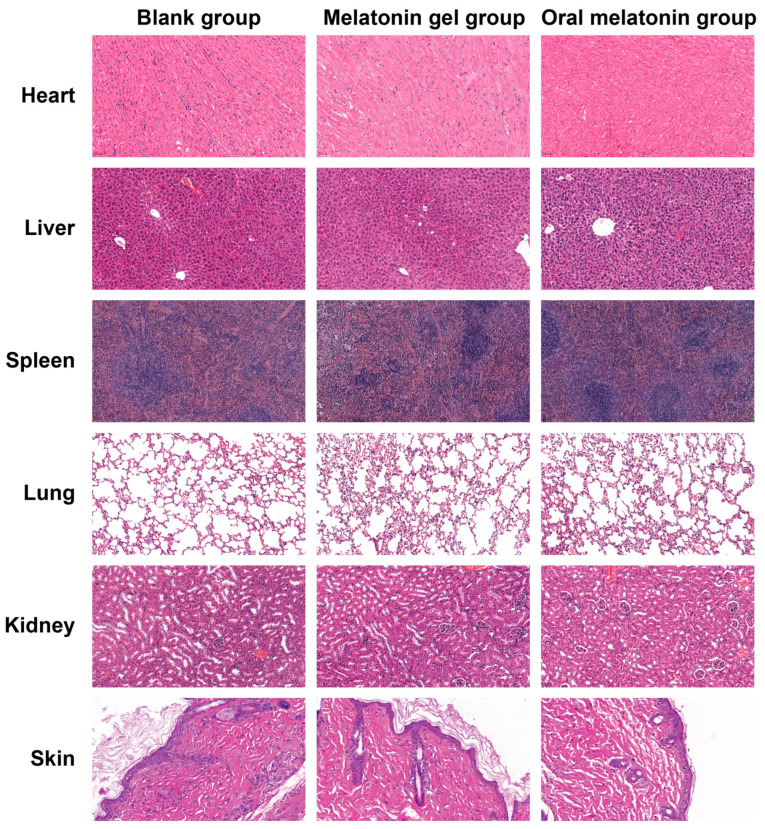
Histological sections of major organs and the application site in mice (H&E staining). Representative optical microscope images of the heart, liver, spleen, lung, kidney and skin tissue from the administration site (from **top** to **bottom**). All images were captured at 200× magnification.

The melatonin-loaded p(NVCL) transdermal gel exhibited excellent safety and tolerability throughout the 28-day administration period. No visible signs of erythema, oedema or other local irritation were observed at the application site, indicating the excellent compatibility of the p(NVCL) matrix and selected transdermal enhancer combination with skin. As shown in Table S3, biochemical analysis after 28 days of continuous application revealed that all measured parameters, including hematological indicators (white blood cell [WBC], red blood cell [RBC], hemoglobin [HGB] and platelets) and biochemical indicators (alanine aminotransferase [ALT], aspartate aminotransferase [AST], alkaline phosphatase, blood urea nitrogen [BUN] and creatinine [Cr]), remained within normal physiological ranges, with no statistically significant differences compared with the blank group (*p* > 0.05). These results indicate that the gel formulation does not exhibit noticeable systemic toxicity and is suitable for long-term applications. Figure 7 presents the histopathological examination results for major organs (heart, liver, spleen, lungs and kidneys) and skin tissue from the application site. After 28 days of treatment, all tissues examined in the melatonin gel group maintained their integrity, with no evidence of inflammatory cell infiltration or tissue damage, consistent with Besag et al.’s conclusions in their systematic review on the safety of long-term melatonin supplementation [46].

### 2.6. In Vivo Biodistribution and Transdermal Absorption Visualization

#### 2.6.1. Synthesis and Characterization of Cy5.5-Melatonin Fluorescent Probe

The Cy5.5-melatonin fluorescent probe was successfully synthesized with a molecular weight of 864.1 Da (Figure S2). Liquid chromatography analysis demonstrated successful synthesis with the target compound, eluting at a retention time of 6.68 min. The chromatographic profile showed a well-defined peak with the maximum intensity reaching 4.8 × 10⁵ arbitrary units, indicating high compound purity and appropriate analytical conditions.

Mass spectrometric analysis revealed a prominent molecular ion peak at *m*/*z* 826.5, which corresponded to the expected molecular ion of the Cy5.5-melatonin conjugate (Figure S3c). The observed mass-to-charge ratio was consistent with the calculated molecular weight, accounting for ionization conditions during the electrospray ionization process.

Fluorescence spectroscopic analysis confirmed that Cy5.5-melatonin exhibited maximum excitation at 680.0 nm with a fluorescence intensity of 120,843.3 units (Figure S3a). When excited at 680.0 nm, the compound displayed maximum emission at 706.0 nm with a fluorescence intensity of 119,497.9 units (Figure S3b). The Stokes shift was calculated to be approximately 26 nm, indicating excellent spectral separation between excitation and emission maxima. These results confirmed that the conjugation process preserved the photophysical properties of the Cy5.5 fluorophore while maintaining the biological functionality of the melatonin moiety.

#### 2.6.2. In Vivo Near-Infrared Fluorescence Imaging

In vivo fluorescence imaging revealed distinct biodistribution patterns among the three experimental groups six hours post-administration (Figure S4). The negative control group (unlabeled melatonin) showed minimal background fluorescence throughout the body, confirming the specificity of the imaging approach. Mice receiving oral Cy5.5-melatonin exhibited moderate fluorescence signals distributed primarily in the abdominal region, consistent with gastrointestinal absorption and hepatic metabolism pathways.

The transdermal gel group demonstrated the most pronounced and widespread fluorescence distribution patterns. Strong fluorescence signals were observed at the application site on the dorsal neck region, indicating successful gel adhesion and initial drug release. Notably, fluorescence signals were detected throughout multiple anatomical regions, including the thoracic and abdominal cavities, suggesting the effective systemic absorption and distribution of the fluorescently labeled melatonin through the transdermal route.

#### 2.6.3. Biodistribution Analysis and Transdermal Absorption Confirmation

**Figure 8:**
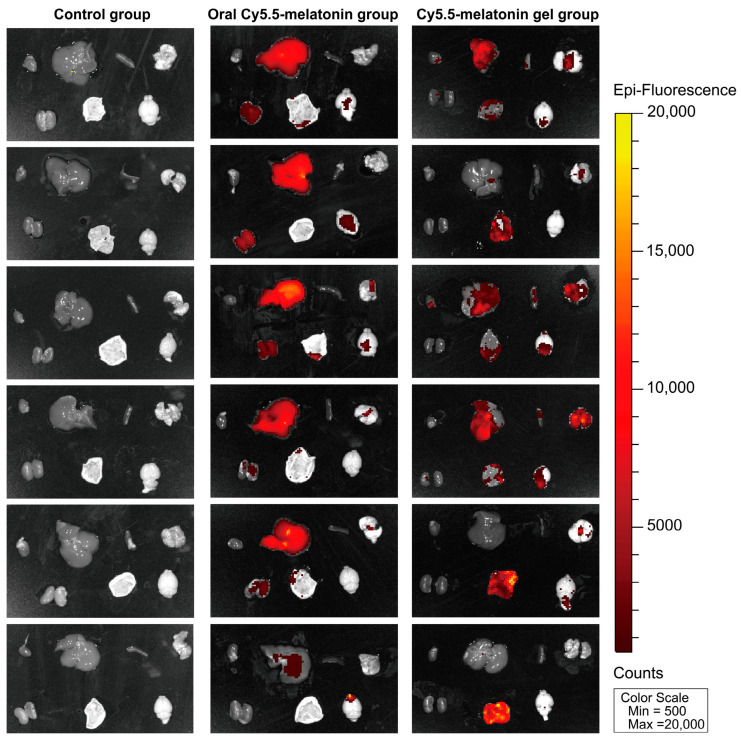
Ex vivo organ biodistribution analysis of Cy5.5-melatonin. Representative fluorescence images of excised organs including the brain, heart, liver, spleen, lung, kidney, and dorsal skin tissue.

Ex vivo organ analysis provided detailed insights into the biodistribution patterns of Cy5.5-melatonin across different administration routes (Figure 8). The negative control group showed negligible fluorescence in all examined organs, confirming the absence of autofluorescence interference. Organs from mice receiving oral Cy5.5-melatonin displayed moderate fluorescence signals, particularly in metabolically active tissues such as the liver and kidneys. The transdermal gel group exhibited superior organ penetration and distribution compared to oral administration. Fluorescence signals were distributed throughout various organs including the heart, liver, spleen, lung, and kidney tissues, indicating comprehensive systemic bioavailability. This finding was particularly significant given the critical role of melatonin in regulating sleep–wake cycles through central mechanisms.

These results provided direct visual evidence supporting the enhanced transdermal absorption efficiency achieved through the optimized p(NVCL)-based delivery system and composite enhancer formulation. The biodistribution study conclusively demonstrated that the melatonin-loaded p(NVCL) transdermal gel successfully facilitated drug absorption through the skin barrier and achieved effective systemic distribution, providing mechanistic validation for the sleep quality improvements observed in behavioral and physiological assessments.

Collectively, these findings provide comprehensive experimental evidence supporting the clinical potential of the p(NVCL) transdermal gel formulation as a safe and effective sleep quality enhancer. The results of the extensive safety evaluation indicated that the melatonin-loaded p(NVCL) transdermal gel formulation not only effectively improved sleep quality but also exhibited excellent safety and tolerability, making it a suitable non-prescription sleep aid that can meet the increasing demand for accessible solutions to improve sleep quality.

Although our results demonstrate considerable potential for the melatonin-loaded p(NVCL) transdermal gel system, there are several research limitations from a rigorous scientific perspective. First, significant interspecies differences exist between human and rodent skin, particularly in terms of the stratum corneum structure, appendage distribution and transdermal absorption mechanisms. Future research should validate the transdermal parameters through ex vivo Franz diffusion cell techniques using human skin samples to enhance the prediction of the clinical performance. Second, while the morning administration protocol used in this study is appropriate for diurnal rodents, adjustments may be required for nocturnal humans aiming to optimize their chronobiological alignment with endogenous melatonin rhythms. Finally, while our 28-day assessment offers valuable preliminary safety data, more comprehensive long-term safety assessments are needed, particularly concerning the potential effects of feedback inhibition on the hypothalamic–pituitary–pineal axis resulting from prolonged exogenous melatonin supplementation. These considerations will inform the next phase of translational research as we progress towards the clinical application of this promising formulation.

## 3. Conclusions

This investigation demonstrates the development of an improved melatonin delivery technology that addresses several limitations associated with conventional therapeutic approaches. The integration of thermosensitive polymer chemistry with optimized enhancer formulations has resulted in a delivery system that modifies melatonin pharmacokinetics by reducing hepatic metabolism effects while maintaining appropriate safety profiles. The research establishes a systematic development approach that combines polymer design principles, formulation optimization techniques, and comprehensive preclinical evaluation to demonstrate both therapeutic efficacy and safety characteristics. The observed absence of tolerance development and reversible effects upon treatment cessation suggest that this approach may complement endogenous circadian regulation mechanisms rather than disrupting natural sleep processes, which represents a notable advantage over some existing sleep aids.

The practical applications of this work address several important areas within sleep medicine and public health. Healthcare providers face a growing demand for effective sleep interventions as sleep-related disorders become increasingly prevalent across different population groups. This technology offers potential solutions for three key areas: consumer markets seeking non-prescription alternatives to conventional sleep medications, elderly populations experiencing age-related changes in melatonin production who may benefit from enhanced drug delivery methods, and occupational health programs addressing circadian rhythm challenges in shift workers. The demonstrated therapeutic benefits and safety profiles support clinical development potential, with future research focusing on human skin validation and manufacturing optimization to contribute to available sleep quality treatment options.

## 4. Materials and Methods

### 4.1. Materials and Reagents

NVCL (purity ≥ 97%) was purchased from Adamas-beta Technology Co., Ltd. (Shanghai, China). AIBI, N,N-dimethylformamide (DMF), dimethyl sulfoxide, carbopol 940, triethanolamine and propylene glycol were obtained from Shanghai Macklin Bio-chem Co., Ltd. (Shanghai, China). Melatonin (purity ≥ 98%) was purchased from Sigma-Aldrich Co., Ltd. (St. Louis, MO, USA). Tetrahydrofuran, acetonitrile, ether and methanol were purchased from Sinopharm Chemical Reagent Co., Ltd. (Shanghai, China). Ultra-pure water was prepared in our laboratory. All other reagents and solvents were of analytical grade.

### 4.2. Synthesis and Characterisation of p(NVCL)

#### 4.2.1. Polymerization Procedure

The polymerization procedure was optimized based on our research group’s previously established methodology, with key modifications made to achieve molecular weight characteristics suitable for transdermal gel applications [47]. NVCL monomer (10 g) was dissolved in a mixed solvent of deionized water (35 mL) and anhydrous ethanol (15 mL) in a 250 mL three-neck round-bottom flask. Under nitrogen protection, the AIBI initiator (0.01 g) was added to the reaction system, and the pH was adjusted to 7.0 using triethanolamine. After thorough deoxygenation using three freeze–pump–thaw cycles, polymerization was conducted at 70 °C for 6 h under magnetic stirring (300 rpm). Upon completion, the mixture was cooled to room temperature, transferred into a dialysis bag (molecular weight cut-off of 3500 Da) and dialyzed against deionized water for 72 h (with the dialysis solution replaced every 12 h). The product was subsequently lyophilized (−70 °C, 72 h) to obtain p(NVCL).

#### 4.2.2. FTIR Spectroscopic Analysis

The NVCL monomer, p(NVCL) polymer, and melatonin-loaded gel samples (5 mg each) were vacuum-dried and ground into a fine powder. The melatonin-loaded gel sample was freeze-dried at −70 °C for 48 h prior to analysis to remove residual moisture and obtain a dry powder suitable for FTIR characterization. FTIR spectra were acquired using an FTS-6000 spectrometer (Bio-Rad, Hercules, CA, USA), with a wavenumber range of 400–4000 cm^−1^, a scanning rate of 4 cm^−1^/s and a resolution of 8 cm^−1^. Each spectrum was collected as an average of 32 scans to ensure an adequate signal-to-noise ratio. Spectra data were processed using OriginPro 9.0 software for baseline correction and peak identification.

#### 4.2.3. LCST Determination

The phase transition temperature (LCST) of the p(NVCL) samples was determined using a DSC 214 Polyma differential scanning calorimeter (NETZSCH, Selb, Germany). Aqueous sample solutions (5 mg/mL) were prepared and heated from 20 °C to 45 °C at a constant scanning rate of 2 °C/min under a nitrogen atmosphere. The LCST value was identified as the intersection point between the heat flow curve and the baseline using Universal Analysis software. Universal Analysis Software (version v2.0).

#### 4.2.4. GPC Analysis

The p(NVCL) samples (5 mg) were dissolved in tetrahydrofuran at a concentration of 4 mg/mL. The analysis was conducted using a Waters GPC system (Waters Corporation, Milford, MA, USA) equipped with a differential refractive index detector and Styragel HR series columns. Tetrahydrofuran was employed as the mobile phase, with a flow rate of 1.0 mL/min and a column temperature of 35 °C. M_w_, M_n_ and PDI were determined.

#### 4.2.5. PXRD Analysis

PXRD patterns were obtained using a Rigaku Miniflex 600 X-ray (Rigaku Corporation, Tokyo, Japan) diffractometer equipped with Cu Kα radiation (λ = 1.5406 Å). Samples were analyzed over a 2θ range of 5–50° with a scanning rate of 2°/min and step size of 0.02°. The operating voltage and current were set at 40 kV and 15 mA, respectively. Three samples were analyzed: pure melatonin, p(NVCL) polymer, and the melatonin-loaded gel formulation. All samples were ground to fine powder and mounted on sample holders for analysis.

### 4.3. Preparation of Melatonin-Loaded p(NVCL) Transdermal Gel

The melatonin-loaded p(NVCL) transdermal gel was prepared using a two-step procedure. First, to establish the gel matrix, p(NVCL) (25%, *w*/*w*) was dispersed in pre-cooled deionized water at 4 °C and uniformly stirred at 300 rpm using a digitally controlled stirrer (Scilogex LLC, Beijing, China) for 6 h. The resulting mixture was then allowed to stand overnight at 4 °C. HPMC (1.5%, *w*/*w*) was gradually added to the solution and stirred until fully dissolved. Separately, carbopol 940 (0.5%, *w*/*w*) was dispersed in deionized water, stirred at low speed (150 rpm) for 30 min until fully swollen and adjusted to pH 7.0 using triethanolamine. Second, involving melatonin loading and homogenization, melatonin (3%, *w*/*w*) was dissolved in ethanol (8%, *w*/*w*) and ultrasonicated at 40 °C for 10 min. The melatonin–ethanol solution was then added dropwise to the p(NVCL)-HPMC mixture at 25 °C, at a controlled rate of 1 mL/min under continuous medium-speed stirring (200 rpm). Subsequently, the carbopol gel base was gradually added to the mixture and stirred for an additional 30 min to achieve a uniform composite gel. The resulting gel was then transferred to a high-speed dispersing homogenizer and processed at 10,000 rpm for 2 min. The prepared gel was left to stand at room temperature for 2 h and then stored in the dark at 4 °C for 24 h. All preparation steps were conducted under light-protective conditions in the dedicated darkroom facility, with melatonin solutions prepared and stored in amber glass vials, and the final gel products maintained in amber containers throughout the equilibration and storage periods.

### 4.4. Determination of Melatonin Content

A UV-2600 spectrophotometer (Shimadzu, Kyoto, Japan) was used to determine the melatonin content in the gel. The melatonin reference standard was first prepared as a 500-μg/mL stock solution in methanol. This stock solution was subsequently diluted with methanol to prepare a series of standard solutions in the concentration range of 10.0–100.0 μg/mL. Absorbance readings were recorded at the maximum absorption wavelength of 223 nm, and a standard curve was established with the following equation: A = 0.0213C − 0.0024 (r = 0.98), where A represents absorbance and C represents the melatonin concentration (μg/mL). For sample analysis, approximately 1.0 g of gel was transferred into a 25-mL volumetric flask, extracted with methanol using ultrasonication for 10 min, diluted to volume with methanol and centrifuged at 4000 rpm for 10 min. The absorbance of the resulting supernatant was measured, and the melatonin content was quantified using a standard curve. The melatonin loading and encapsulation efficiency were calculated using the following formulas: melatonin loading (%) = (measured drug mass/total gel mass) × 100; encapsulation efficiency (%) = (measured drug mass/theoretical drug content) × 100. Each sample was analyzed in triplicate, and the mean value and standard deviation (SD) were calculated.

### 4.5. Optimisation of Transdermal Permeation Enhancers Transdermal Permeation Enhancer Optimization Methodology

#### 4.5.1. Franz Diffusion Cell Setup

A modified Franz vertical diffusion cell system (Model C0310, Shanghai Kai Technology Co., Ltd., Shanghai, China) was used to evaluate the in vitro transdermal permeation performance of the melatonin-loaded p(NVCL) gel. SD rats (200 ± 20 g) were shaved in the dorsal neck region 24 h before sacrifice. Following euthanasia, intact skin from the shaved area was excised, the underlying subcutaneous tissue and fat were removed and the skin was washed with physiological saline and stored at −20 °C until further use. Before the experiments, skin samples were brought to room temperature and mounted on the diffusion cell, with the stratum corneum oriented towards the donor compartment. The effective permeation area was 3.14 cm^2^, with 15 mL of PBS buffer (pH 7.4) as the receptor fluid, maintained at 32 ± 0.5 °C in a thermostatically controlled water bath. Continuous stirring at 600 rpm was applied to ensure homogeneity. Transdermal flux values were calculated as the ratio of 24 h cumulative permeation to the experimental duration. Results were expressed in μg/cm^2^/h to evaluate the drug permeation rates.

#### 4.5.2. Screening of Single Permeation Enhancers Single Permeation Enhancer Screening Protocol

The swelling method was employed to incorporate varying concentrations of individual permeation enhancers into the p(NVCL) gel base, thereby generating distinct formulations. In the first set, menthol was added at concentrations of 1%, 2%, 3%, 4% and 5% (*w*/*w*). A separate set of formulations was prepared with borneol at 2%, 4%, 6% and 8% (*w*/*w*). In the third set, azone was incorporated at concentrations of 1%, 2%, 3%, 4% and 5% (*w*/*w*). Each gel formulation (0.5 g) was uniformly applied to the donor compartment of a Franz diffusion cell. After 24 h, the samples were collected and analyzed using high-performance liquid chromatography to determine the cumulative amount of melatonin permeated. A formulation without any enhancer served as the blank group. All experiments were conducted in triplicate. Both cumulative permeation and transdermal flux data were reported as mean ± SD.

#### 4.5.3. Composite Enhancer System Development Protocol

Based on the results of the single enhancer screening, an L9(3^3^) orthogonal experimental design was employed to optimize the enhancer system. Azone, menthol and borneol were selected as the experimental factors, each tested at three concentration levels: azone (1%, 3% and 5% *w*/*w*), menthol (1%, 3% and 5% *w*/*w*) and borneol (2%, 4% and 6% *w*/*w*). Each formulation underwent a 24 h permeation experiment in the Franz diffusion cell, with cumulative melatonin permeation over 24 h serving as the evaluation index. Transdermal flux calculations were performed to compare the permeation rates among different enhancer combinations. All experiments were conducted in triplicate, and the results were expressed as mean ± SD.

### 4.6. Characterisation of Melatonin-Loaded p(NVCL) Transdermal Gel

#### 4.6.1. Physicochemical Property Evaluation

A SevenCompact™ S210 pH meter (Mettler Toledo, Greifensee, Switzerland) was used to measure the pH of the melatonin-loaded p(NVCL) transdermal gel. The moisture content was quantified using precise weighing combined with Karl Fischer titration. Gel samples (2.0 g) were accurately weighed into pre-dried aluminum containers and dried under vacuum at 60 °C until a constant weight was achieved. Mass readings were taken at 2 h intervals, and drying was considered complete when the difference between successive measurements was less than 0.2%. The moisture content (%) was calculated using the following formula: (W_1_ − W_2_)/W_1_ × 100. Here, W_1_ and W_2_ denote the initial and dried sample masses, respectively. The microstructure of the gel was characterized using cryogenic scanning electron microscopy (Cryo-SEM). Freshly prepared gel samples were rapidly frozen in liquid nitrogen, fractured at −140 °C, sputter-coated with gold and examined at −90 °C to observe their internal morphology.

#### 4.6.2. Rheological Characterisation

A DHR-2 rheometer (TA Instruments, New Castle, DE, USA) was used to determine the rheological properties of the gel. Samples were loaded between 40 mm titanium parallel plates with a fixed gap of 1.0 mm. To determine the linear viscoelastic region, strain sweep tests were first performed over a range of 0.01–100%. Subsequently, frequency sweep tests were conducted within the established linear viscoelastic region. Temperature sweep tests were conducted at 1 Hz and 1% strain across a temperature range of 15–45 °C, with a controlled heating rate of 2 °C/min. Throughout the test, the G′, G″ and complex viscosity (η*) were recorded.

#### 4.6.3. Thixotropic Property Assessment

A Discovery HR-3 hybrid rheometer (TA Instruments, New Castle, DE, USA) was used to evaluate the thixotropic properties of the gel. Using a cone-plate measuring system (40 mm diameter, 2° cone angle), a three-step shear test was conducted at 25 °C: low shear phase (0.1 s^−1^, 60 s), high shear phase (100 s^−1^, 60 s) and low shear recovery phase (0.1 s^−1^, 180 s). The thixotropic recovery index was calculated as follows: η_3_/η_1_ × 100%, where η_3_ is the steady-state viscosity in the recovery phase and η_1_ is the steady-state viscosity in the initial low shear phase. In addition, a TA.XT plus texture analyzer (Stable Micro Systems, Godalming, UK) was used to measure the extensibility properties of the gel, using a TTC spreadability fixture with the following test conditions: pre-test speed 2 mm/s, trigger force 5 g, test speed 3 mm/s, return speed 10 mm/s and displacement 25 mm.

#### 4.6.4. Stability Studies Stability Testing Protocol

The prepared melatonin-loaded p(NVCL) gel was transferred into amber glass bottles, sealed and stored under three different conditions, i.e., at room temperature (25 ± 2 °C, relative humidity 60 ± 5%), low temperature (4 ± 2 °C, relative humidity 60 ± 5%) and accelerated conditions (40 ± 2 °C, relative humidity 75 ± 5%). Samples were taken at 0, 1 and 6 months to examine the appearance, pH, viscosity, extensibility, moisture content and melatonin concentration. The drug retention rate (%) was calculated using the following formula: (C_t_/C_0_) × 100%. Here, C_t_ represents the drug content at time t and C_0_ denotes the initial drug content.

### 4.7. Evaluation of the Sleep Quality Effects and Safety of Melatonin-Loaded p(NVCL) Transdermal Gel

#### 4.7.1. Animal Grouping and Administration Protocol

SPF-grade male Kunming mice (19–23 g, n = 90) were randomly divided into a blank group, melatonin gel group and oral melatonin group after 1 week of adaptation, with 30 mice per group. The animal experimental protocol was approved by the Experimental Animal Ethics Committee of Yunnan University of Chinese Medicine (approval number: R-062021020). Mice in the melatonin gel group (19–23 g) had their dorsal neck region shaved (1 × 1 cm area) and received 0.2 g melatonin-loaded p(NVCL) transdermal gel daily at 9:00 AM. Mice in the oral melatonin group received melatonin (20 mg/kg) through gavage daily.

#### 4.7.2. Behavioral Assessment of Sleep Promotion Behavioral Assessment Methodology

From each group of 30 mice, 12 were randomly selected for an evaluation of their sleep parameters and quality. Initially, motor parameters were recorded using an XR-ZFT small animal behavioral analysis system for 0–6 h after administration. These parameters included the total activity distance, time spent in the central area and rearing frequency. Subsequently, pentobarbital-induced sleep tests were conducted to assess the sleep-promoting effects of melatonin. Two hours after the gel application or oral administration, the mice received intraperitoneal injections of sodium pentobarbital (45 mg/kg). The sleep latency (the time from injection to the loss of the righting reflex) and sleep duration (the time from loss to recovery of the righting reflex) were recorded.

#### 4.7.3. Electroencephalographic Sleep Structure Analysis Electroencephalographic Monitoring Protocol

The 12 mice described earlier underwent the surgical implantation of EEG electrodes for sleep state monitoring. After anesthesia with sodium pentobarbital (Sigma-Aldrich, St. Louis, MO, USA, 50 mg/kg), the mice were fixed in a stereotaxic apparatus, and stainless-steel screw electrodes were implanted in the cerebral cortex based on the coordinates of the mouse brain stereotaxic atlas. Reference electrodes were placed on the frontal bone and ground electrodes on the occipital region. All electrodes were connected to a miniature connector fixed to the skull using dental cement. Penicillin was administered post-surgery to prevent infection, and the mice were allowed a 7-day recovery period before testing. On day 28 of administration, the sleep quality of the mice was evaluated. A BL-420F biological function experimental system continuously recorded EEG signals for 24 h through the implanted electrodes. The mice were connected to the recording system and allowed to move freely in a sound-attenuated, temperature-controlled environment. The EEG signals were amplified, band-pass filtered and digitized at a 200-Hz sampling rate. Based on the EEG waveform characteristics, the sleep–wake states were classified as WAKE, NREM sleep and REM sleep. The time distribution and transition patterns of each state were then analyzed.

#### 4.7.4. Neurochemical Mechanism Investigation

On days 14, 28 and 42 of administration (i.e., 14 days after cessation), six mice were randomly selected from each group and humanely sacrificed. Blood samples were collected for biochemical analysis. ELISA was used to determine the serum levels of melatonin and 5-HT.

#### 4.7.5. Safety and Tolerability Assessment

The remaining 12 mice in each group, which had not undergone electrode implantation, were used for the evaluation of in vivo tolerability. These mice received only the assigned treatment, which was consistent with their group allocation. The application site was examined daily for signs of local irritation, such as erythema and oedema. Subsequently, all 12 mice were euthanized. Blood samples, major organs (heart, liver, spleen, lungs and kidneys) and skin tissue from the application site were collected for biochemical and histopathological analyses. Hematological and biochemical parameters, including the WBC count, RBC count, HGB, BUN, Cr, AST and ALT, were measured using an automated biochemical analyzer (AU5400, Olympus, Tokyo, Japan), strictly following the manufacturer’s reagent kit instructions. Tissue samples from the heart, liver, spleen, lung, kidney and application site skin were fixed with 4% paraformaldehyde, routinely embedded in paraffin, sectioned and stained with haematoxylin and eosin. Histological examination was conducted using an optical microscope to assess potential pathological changes.

### 4.8. Synthesis of Cy5.5-Melatonin Fluorescent Probe and In Vivo Imaging

#### 4.8.1. Chemical Synthesis of Cy5.5-Melatonin

Cy5.5-melatonin was synthesized through a multi-step approach. 5-hydroxytryptamine hydrochloride (1.0 eq.) was dissolved in DMF and treated with acetic anhydride (2.0 eq.) and DIPEA (3.0 eq.) at room temperature for 0.5 h, followed by column chromatography purification.

The resulting product was dissolved in DMF and treated with Boc anhydride (3.0 eq.) and DMAP (0.5 eq.) at room temperature for 0.5 h. After column chromatography purification, the intermediate was dissolved in DMF and combined with N-Boc-bromoethylamine (3.0 eq.), cesium carbonate (5.0 eq.), and potassium iodide (0.5 eq.) at 60 °C for 3 h. The reaction mixture was concentrated, extracted with dichloromethane, washed with 5% citric acid solution and saturated brine, then dried over sodium sulfate.

Deprotection was achieved using TFA/DCM (1:2, 2 mL) at room temperature for 0.5 h to obtain Melatonin-NH_2_. Final coupling was performed with Cy5.5-COOH (0.5 eq.), DIPEA (5.0 eq.), and PyBOP (1.5 eq.) at room temperature for 0.5 h, followed by column chromatography purification.

#### 4.8.2. Analytical Characterization

Analysis was performed using an LC-20AD XR system coupled to an API3200 mass spectrometer (AB Sciex, Framingham, MA, USA) with a Shim-pack GIST-HP C18 column (3 μm, 150 mm × 2.1 mm). The mobile phase consisted of methanol and water (both containing 0.1% formic acid) with a gradient from 40% to 100% methanol over 4 min at a 0.2 mL/min flow rate. Detection was performed at 660 nm with a 10 μL injection volume. Mass spectrometry was performed using positive ion mode with optimized parameters: curtain gas of 15 units, ion spray voltage of 5500 V, and source temperature of 500 °C.

Measurements were conducted using an RF-6000 fluorescence spectrometer (Shimadzu Corporation, Kyoto, Japan) with 5.0 nm bandwidths and a 6000 nm/min scanning speed. Excitation spectra were recorded from 600–800 nm with emission fixed at 706 nm. Emission spectra were recorded from 600 to 800 nm with excitation at 680 nm.

#### 4.8.3. In Vivo Near-Infrared Fluorescence Imaging Protocol

SPF-grade male Kunming mice (19–23 g, n = 18) were randomly divided into three groups with 6 mice per group after 1 week of adaptation. All mice had their dorsal and ventral regions shaved to prevent hair interfering with imaging. The control group received oral unlabeled melatonin (20 mg/kg) as a negative control. The oral Cy5.5-melatonin group received oral Cy5.5-melatonin (equivalent to 20 mg/kg melatonin). The transdermal Cy5.5-melatonin gel group application received a topical application of 0.2 g Cy5.5-melatonin loaded p(NVCL) transdermal gel to the shaved dorsal neck area (1 × 1 cm).

Six hours after administration, mice were anesthetized with isoflurane inhalation anesthesia and subjected to in vivo fluorescence imaging using an IVIS Spectrum imaging system (PerkinElmer, Waltham, MA, USA). Near-infrared fluorescence images were acquired with excitation at 680 nm and emission at 720 nm. Imaging parameters were set with a binning of 4, a field of view of 22.8 cm, and f-stop of 2. The exposure time was optimized for each imaging session to achieve an optimal signal-to-noise ratio. Following in vivo imaging, mice were euthanized and major organs including the brain, heart, liver, spleen, lung, kidney, and dorsal skin tissue from the application site were carefully excised. The ex vivo fluorescence imaging of individual organs was performed using identical imaging parameters.

### 4.9. Statistical Analysis

All experimental data are expressed as mean ± SD. Statistical analyses were conducted using SPSS software (version 25.0). Intergroup comparisons were conducted using one-way analysis of variance, followed by the least significant difference test for multiple comparisons. A *p* value of <0.05 was considered statistically significant. Graphs were generated using GraphPad Prism (version 10.0).

## Data Availability

All data supporting the findings of this study are included in the article and its Supplementary Materials.

## Supplementary Materials

The following supporting information is available for download at https://www.mdpi.com/article/10.3390/gels11060435/s1. Figure S1: Gel permeation chromatography (GPC) analysis of the p(NVCL) polymer. The blue curve represents the differential molecular weight distribution (dw/dlogM), whereas the red curve indicates the cumulative molecular weight distribution as a percentage (%). Figure S2: Chemical structure of the synthesized Cy5.5-melatonin fluorescent probe. Figure S3: Spectroscopic and chromatographic characterization of Cy5.5-melatonin fluorescent probe. (a) Excitation spectrum of Cy5.5-melatonin fluorescent probe. (b) Emission spectrum of Cy5.5-melatonin fluorescent probe. (c) Liquid chromatography-mass spectrometry analysis of Cy5.5-melatonin. Figure S4: In vivo near-infrared fluorescence imaging of mice following different melatonin administration routes. Representative images show biodistribution patterns at 6 h post-administration across three treatment groups. Each row represents individual animals (n = 6 per group). Table S1: Stability parameters of the melatonin-loaded p(NVCL) transdermal gel under various storage conditions (4 °C, 25 °C and 40 °C) over storage durations of 0, 1, 3 and 6 months. Data are expressed as mean ± standard deviation (SD) (n = 3 per group). Table S2: Transdermal flux values of melatonin-loaded p(nvcl) gel with different permeation enhancer systems. Table S3: Hematological and biochemical parameters in mice following 28 days of administration, comparing the blank group, melatonin gel group and oral melatonin group. Data are expressed as mean ± SD (n = 12 per group).
