# Supplementary material for: Effects of Melatonin-Loaded Poly(N-vinylcaprolactam) Transdermal Gel on Sleep Quality
Source: Gels. 2025 Jun 5;11(6):435. doi: 10.3390/gels11060435 (PMC12191773; doi:10.3390/gels11060435)
Supplement: Supplementary file 1 [file gels-11-00435-s001.zip › gels-3615469-supplementary.pdf]

Article

Effects of Melatonin-Loaded Poly(N-vinylcaprolactam) Transdermal Gel on Sleep Quality

Wei Zhao [1,2,3,4,†], Fengyu Wang [2,†], Liying Huang [2], Bo Song [2], Junzi Wu [2,3], Yongbo Zhang [2], Wuyi Du [3,4], Yan Li [1,3,4,*] and Sen Tong [2,*]

[1] Faculty of Life Science and Technology, Kunming University of Science and Technology, Kunming 650500, China; zhaowei@ynucm.edu.cn
[2] Yunnan Key Laboratory of Integrated Traditional Chinese and Western Medicine for Chronic Disease in Prevention and Treatment, Yunnan University of Chinese Medicine, Kunming 650500, China; wangfengyu@ynucm.edu.cn (F.W.); huangliying1609@163.com (L.H.); ynkmsongbo6@126.com (B.S.); xnfz@ynucm.edu.cn (J.W.); nameiszhang6896@163.com (Y.Z.)
[3] Department of Geriatrics, The First People's Hospital of Yunnan Province, Kunming 650032, China; 15587201738@163.com
[4] Medical School, Kunming University of Science and Technology, Kunming 650500, China
* Correspondence: liyanken@126.com (Y.L.); tongsen@ynucm.edu.cn (S.T.)
† These authors contributed equally to this work.

The file includes: **Figures S1-S4 and Tables S1-S3.**

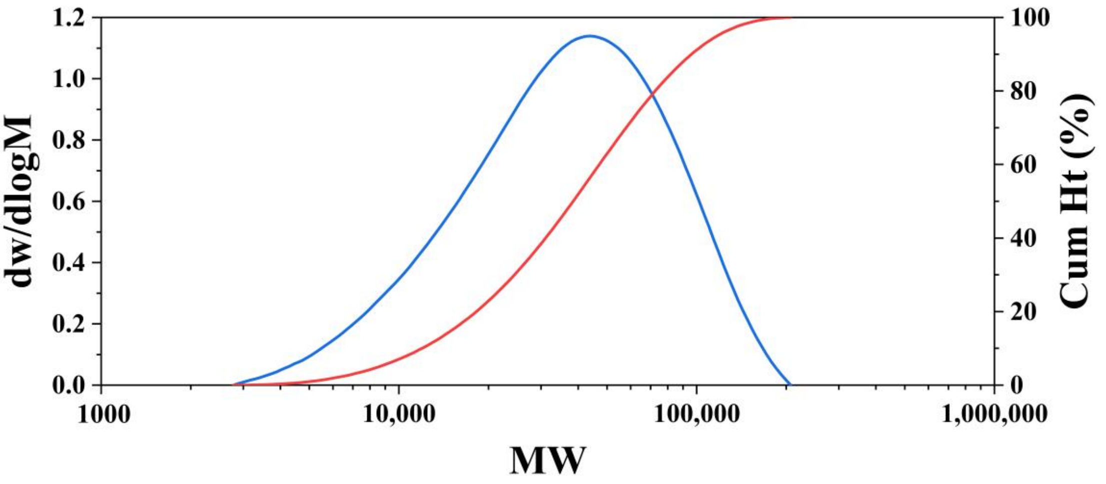

Figure S1. Gel permeation chromatography (GPC) analysis of the p(NVCL) polymer. The blue curve represents the differential molecular weight distribution (dw/dlogM), while the red curve indicates the cumulative molecular weight distribution percentage (%).

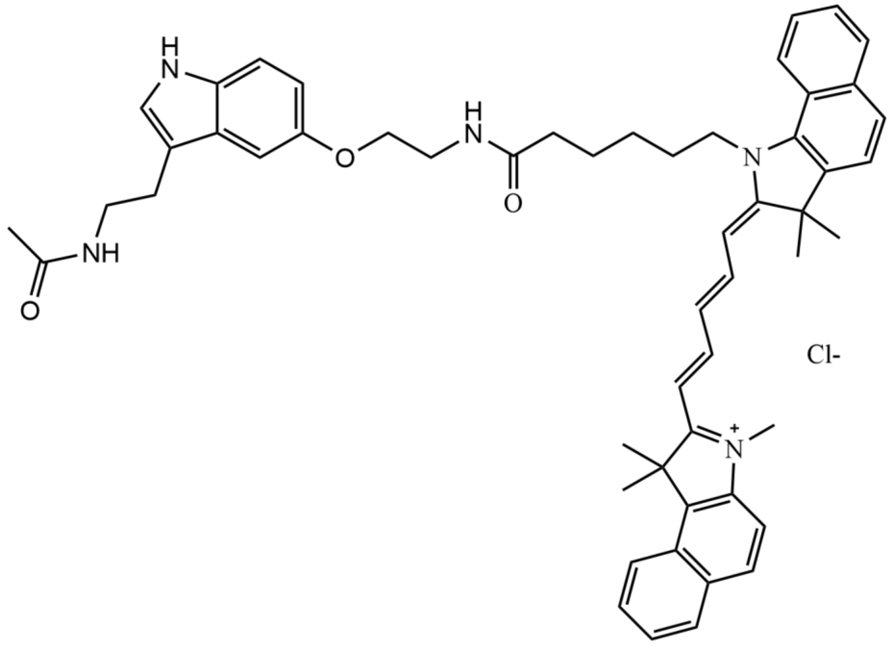

Mw:864.1

Figure S2. Chemical structure of the synthesized Cy5.5-melatonin fluorescent probe.

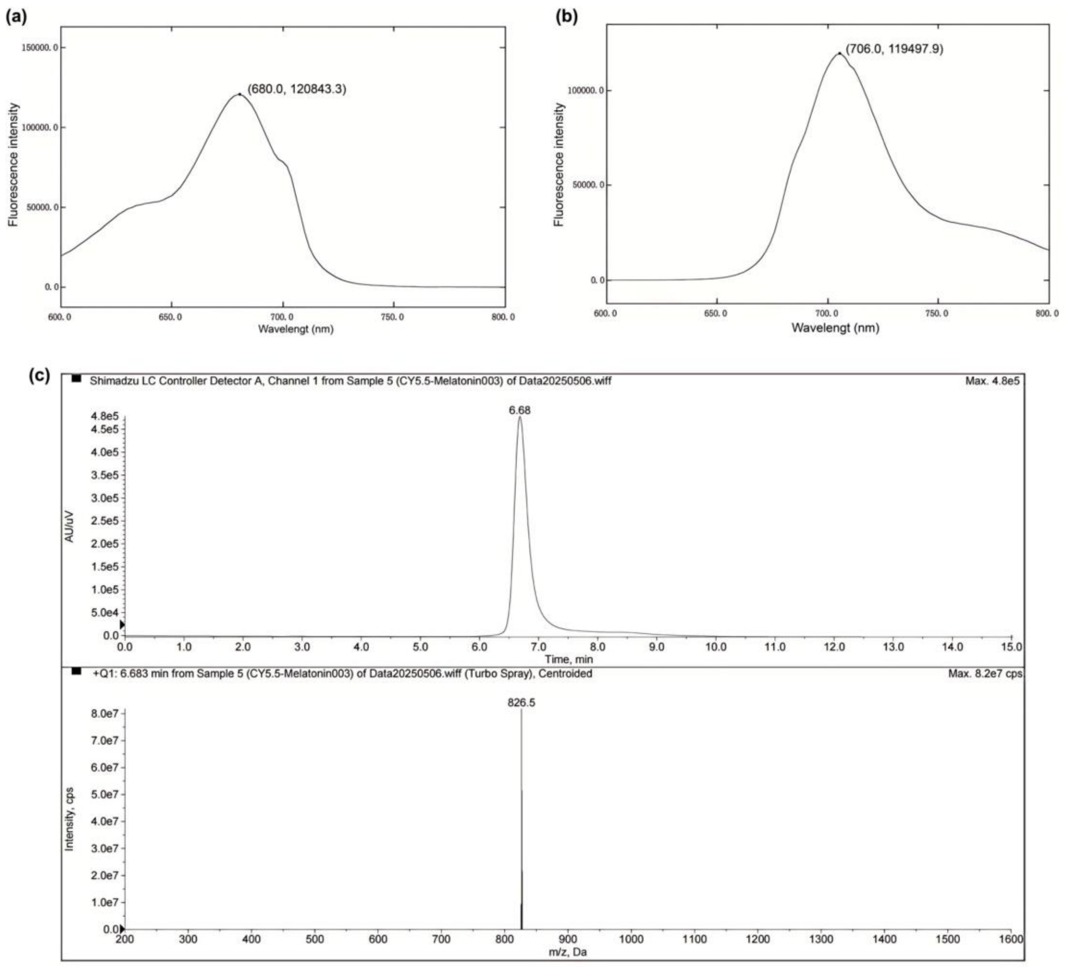

Figure S3. Spectroscopic and chromatographic characterization of Cy5.5-melatonin fluorescent probe. (a) Excitation spectrum of Cy5.5-melatonin fluorescent probe.(b) Emission spectrum of Cy5.5-melatonin fluorescent probe. (c) Liquid chromatography-mass spectrometry analysis of Cy5.5-melatonin.

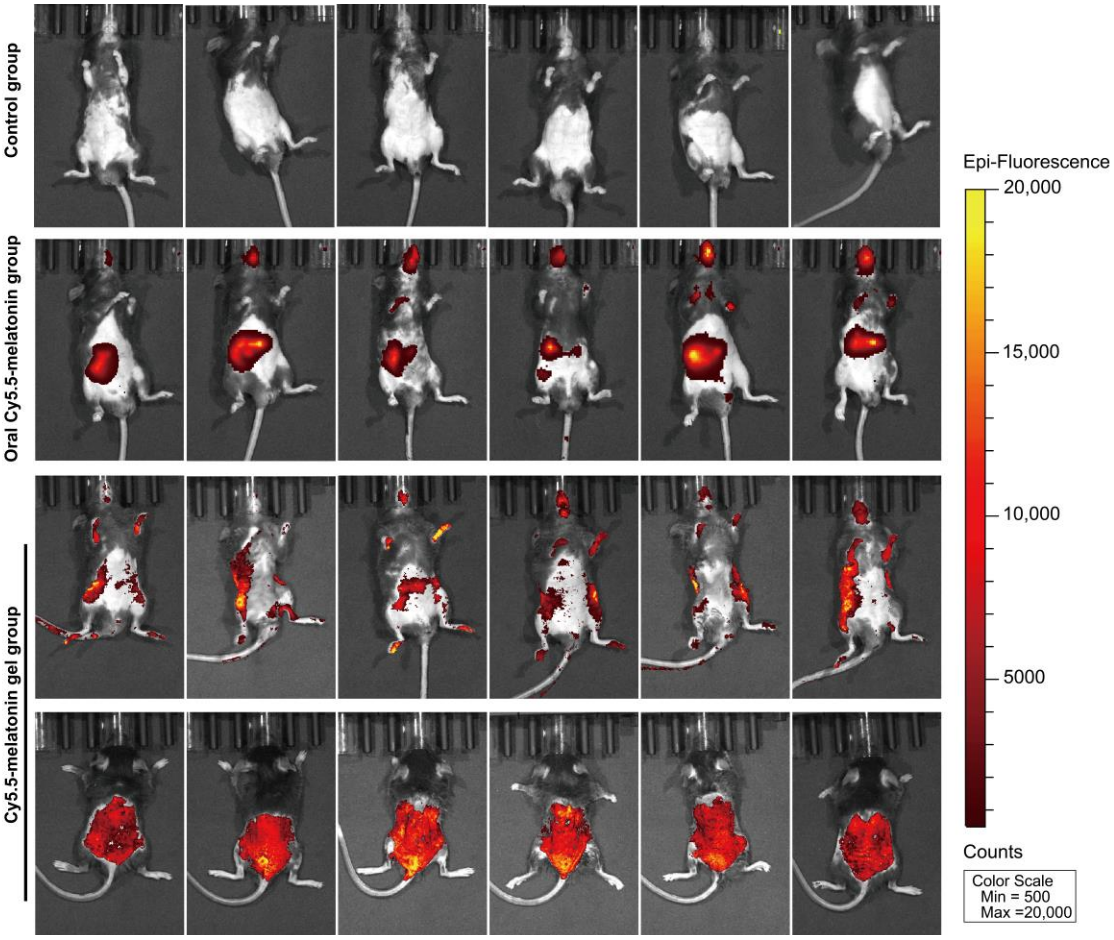

Figure S4. In vivo near-infrared fluorescence imaging of mice following different melatonin administration routes. Representative images show biodistribution patterns at 6 hours post-administration across three treatment groups. Each row represents individual animals (n=6 per group).

Table S1. Stability parameters of melatonin-loaded P(NVCL) transdermal gel at different storage conditions (4°C, 25°C, 40°C) over storage periods of 0, 1, 3, and 6 months. Data are presented as mean ± standard deviation (n=3 per group).

Storage conditions (°C)	Time (month)	Appearance	pH	Moisture content (%)	Viscosity η* (Pa·s)	Extension area (cm²/g)	Drug content retention rate (%)
4°C	0	Transparent and uniform	6.9±0.2	63.5±1.5	46.5±1.8	11.8±0.5	100.0±0.0
4°C	1	Transparent and uniform	6.9±0.2	63.4±1.5	46.4±1.7	11.8±0.5	99.5±0.8
4°C	3	Transparent and uniform	6.9±0.2	63.2±1.6	46.1±1.8	11.7±0.5	98.6±1.0
4°C	6	Transparent and uniform	6.8±0.2	62.9±1.6	45.8±1.8	11.6±0.5	97.4±1.2
25°C	0	Transparent and uniform	6.9±0.2	63.5±1.5	46.5±1.8	11.8±0.5	100.0±0.0
25°C	1	Transparent and uniform	6.9±0.2	63.2±1.6	46.2±1.7	11.7±0.5	99.2±0.9
25°C	3	Transparent and uniform	6.8±0.2	62.8±1.6	45.8±1.8	11.6±0.6	97.6±1.1
25°C	6	Transparent and uniform	6.8±0.3	62.4±1.7	45.2±1.9	11.4±0.6	95.8±1.3
40°C	0	Transparent and uniform	6.9±0.2	63.5±1.5	46.5±1.8	11.8±0.5	100.0±0.0
40°C	1	Transparent and uniform	6.8±0.2	62.8±1.6	45.6±1.9	11.4±0.6	98.2±1.3
40°C	3	Transparent and uniform	6.7±0.3	62.0±1.7	44.8±2.0	11.1±0.7	95.5±1.5
40°C	6	Transparent and uniform	6.6±0.3	61.2±1.8	43.9±2.1	10.8±0.7	93.2±1.6

Table S2. Transdermal flux values of melatonin-loaded p(nvcl) gel with different permeation enhancer systems

Formulation	Transdermal Flux (μg/cm²/h)	Enhancement Ratio*
Single Permeation Enhancers		
Blank group	6.53 ± 0.35	1.0
1% Menthol	9.81 ± 0.51	1.5
2% Menthol	12.44 ± 0.65	1.9
3% Menthol	14.93 ± 0.77	2.3
4% Menthol	15.23 ± 0.80	2.3
5% Menthol	15.37 ± 0.81	2.4
2% Borneol	7.90 ± 0.43	1.2
4% Borneol	10.23 ± 0.56	1.6
6% Borneol	12.37 ± 0.66	1.9
8% Borneol	13.14 ± 0.69	2.0
1% Azone	11.93 ± 0.63	1.8
2% Azone	14.41 ± 0.76	2.2
3% Azone	17.73 ± 0.93	2.7
4% Azone	17.84 ± 0.94	2.7
5% Azone	17.90 ± 0.95	2.7
Composite Permeation Enhancers		
1% Azone + 1% Menthol + 2% Borneol	16.10 ± 0.85	2.5
1% Azone + 2% Menthol + 4% Borneol	17.74 ± 0.94	2.7
1% Azone + 3% Menthol + 6% Borneol	19.52 ± 1.03	3.0
2% Azone + 1% Menthol + 4% Borneol	21.36 ± 1.12	3.3
2% Azone + 2% Menthol + 6% Borneol	23.55 ± 1.23	3.6
2% Azone + 3% Menthol + 2% Borneol	20.78 ± 1.09	3.2
3% Azone + 1% Menthol + 6% Borneol	24.43 ± 1.28	3.7
3% Azone + 2% Menthol + 2% Borneol	21.91 ± 1.15	3.4
3% Azone + 3% Menthol + 4% Borneol	25.66 ± 1.35	3.9

*Enhancement ratio calculated relative to blank group. Data are expressed as mean ± standard deviation (n = 3). Transdermal flux was calculated by dividing 24-hour cumulative permeation by experimental duration.

Table S3. Hematological and biochemical parameters in mice after 28-day administration comparing blank group, melatonin gel group, and oral melatonin group. Data are presented as mean ± standard deviation (n=12 per group).

Serum indicators (units)	blank group	melatonin gel group	oral melatonin group
WBC (×10^9/L)	8.2±0.7	8.3±0.7	8.5±0.7
RBC (×10^{12}/L)	9.8±0.8	9.7±0.8	9.5±0.8
HGB (g/L)	152.5±12.8	151.6±12.7	149.8±12.5
PLT (×10^9/L)	925.6±78.5	928.5±78.8	935.2±79.5
ALT (U/L)	45.6±3.8	45.8±3.8	46.5±3.9
AST (U/L)	128.5±10.8	126.4±10.6	127.2±10.7
ALP (U/L)	156.4±13.2	155.8±13.1	157.5±13.3
BUN (mmol/L)	8.2±0.7	8.3±0.7	8.5±0.7
Cr (μmol/L)	32.5±2.7	32.8±2.8	33.5±2.8
